# Plasma proteomic profile of age, health span, and all‐cause mortality in older adults

**DOI:** 10.1111/acel.13250

**Published:** 2020-10-22

**Authors:** Sanish Sathyan, Emmeline Ayers, Tina Gao, Erica F. Weiss, Sofiya Milman, Joe Verghese, Nir Barzilai

**Affiliations:** ^1^ Department of Neurology Albert Einstein College of Medicine Bronx NY USA; ^2^ Institute for Aging Research, Department of Medicine Albert Einstein College of Medicine Bronx NY USA; ^3^ Department of Genetics Albert Einstein College of Medicine Bronx NY USA

**Keywords:** aging, proteomics, SomaScan^®^ assay, weighted gene co‐expression network analysis

## Abstract

Aging is a complex trait characterized by a diverse spectrum of endophenotypes. By utilizing the SomaScan^®^ proteomic platform in 1,025 participants of the LonGenity cohort (age range: 65–95, 55.7% females), we found that 754 of 4,265 proteins were associated with chronological age. Pleiotrophin (PTN; β[SE] = 0.0262 [0.0012]; *p* = 3.21 × 10^−86^), WNT1‐inducible‐signaling pathway protein 2 (WISP‐2; β[SE] = 0.0189 [0.0009]; *p* = 4.60 × 10^−82^), chordin‐like protein 1 (CRDL1; β[SE] = 0.0203[0.0010]; *p* = 1.45 × 10^−77^), transgelin (TAGL; β[SE] = 0.0215 [0.0011]; *p* = 9.70 × 10^−71^), and R‐spondin‐1(RSPO1; β[SE] = 0.0208 [0.0011]; *p* = 1.09 × 10^−70^), were the proteins most significantly associated with age. Weighted gene co‐expression network analysis identified two of nine modules (clusters of highly correlated proteins) to be significantly associated with chronological age and demonstrated that the biology of aging overlapped with complex age‐associated diseases and other age‐related traits. The correlation between proteomic age prediction based on elastic net regression and chronological age was 0.8 (*p* < 2.2E−16). Pathway analysis showed that inflammatory response, organismal injury and abnormalities, cell and organismal survival, and death pathways were associated with aging. The present study made novel associations between a number of proteins and aging, constructed a proteomic age model that predicted mortality, and suggested possible proteomic signatures possessed by a cohort enriched for familial exceptional longevity.

## Graphical abstract

Age‐associated proteomic changes studied using SomaScan assay showed a plethora of novel proteins and pathways to be associated with aging in older adults. Predicted age using elastic net regression captured mortality better than actual chronological age. Clusters of highly correlated proteins associated with chronological age were also associated with diverse phenotypes and traits giving clues for shared biology.

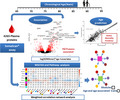

## 1 INTRODUCTION

Aging is a multifactorial phenotype characterized by physiological changes with multifaceted effects or alterations causing gradual functional decline in a living organism (López‐Otín et al., 2013). These age‐associated alterations are also risk factors for various complex diseases such as cancer, diabetes, cardiovascular, and neurodegenerative diseases (López‐Otín et al., 2013). Given its complex nature, the biology of aging has not been completely elucidated yet (Partridge, 2010). A number of genes and proteins in diverse biologic pathways have been implicated in influencing aging and longevity (Johnson et al., 1999). Some of the mechanisms that have been repeatedly linked to aging include DNA instability, telomere shortening, environment‐driven epigenetic changes, cellular senescence, and loss of proteostasis (Johnson et al., 1999; López‐Otín et al., 2013).

Research into the biological mechanisms of aging experienced a big leap with the advent of next‐generation sequencing and genotyping technologies in the last decade, though major advances have been lacking mainly due to the complexity of the phenotype and apparent low heritability (Broer & van Duijn, 2015). Other methodologies, such as transcriptomic and epigenetic analysis, have provided additional insights but a comprehensive elucidation of biology of aging remains elusive (Pal & Tyler, 2016; Zierer et al., 2015). Furthermore, since genes, transcripts, and epigenetic modifications represent intermediate steps in regulation, frequently they are not the final determinants of phenotype. Proteins, on the other hand, in many cases represent the end products of epigenetic and transcription regulation, reflecting the integration of system‐wide biological processes. For instance, it was shown in human fibroblasts that 77% of age‐associated changes in cell protein levels were not correlated with gene transcript levels (Waldera‐Lupa et al., 2014). Proteomic research in aging has been lagging mainly due to the lack of advanced platforms that could facilitate discovery. Recently, the highly multiplexed SomaScan assay provided a major breakthrough by offering a tool that can measure thousands of proteins simultaneously in a small sample of blood (Gold et al., 2010). The principle of SOMAmer reagents is based on aptamer technology and uses single‐stranded DNA‐based protein affinity reagents (Gold et al., 2010). Using this novel technology, we aimed to characterize the proteomic signature of aging, including protein clusters that may reflect resilience to aging and different aging phenotypes.

Evidence indicates that chronological age does not directly correlate with the physiologic and functional status of an individual (Anstey et al., 1996). Differential aging is characterized by the body's ability to maintain homeostasis across different organ systems over time, whereas deterioration of this balance can lead to rapid aging with associated decline in function and occurrence of complex age‐associated diseases (Anstey et al., 1996) that reflect the biological age of an organism. We hypothesized that the proteome can capture the biology underlying the physiological age and not simply the chronological age. We tested this hypothesis in a homogenous community‐dwelling cohort of Ashkenazi Jewish older adults in whom ~4,265 plasma proteins were measured by utilizing the SomaScan platform. As part of the study, we aimed to develop an age prediction model based on the proteome and to test whether it predicted mortality. In addition, our cohort was enriched with individuals with familial longevity, with approximately half of the cohort composed of offspring of parents with exceptional longevity who repeatedly demonstrated better health status compared to age‐matched controls (Ayers et al., 2014; Gubbi et al., 2017). Although aging is a major risk factor for many chronic diseases, individuals with exceptional longevity and their offspring often delay the onset of age‐related diseases and syndromes (Andersen et al., 2012; Ismail et al., 2016) despite having similar lifestyle habits to their peers (Gubbi et al., 2017; Rajpathak et al., 2011) suggesting that longevity is at least in part genetically determined and is heritable (Milman & Barzilai, 2016). Thus, our cohort was particularly suitable for identifying the proteomic signature of resilience to aging and we hypothesized that the offspring of parents with longevity will demonstrate a more youthful proteome compared to age‐matched controls.

## 2 RESULTS

### 2.1 Study population

Of the 1,025 eligible individuals with phenotype and proteomic data in the LonGenity cohort, 506 (49.4%) were offspring of parents with exceptional longevity (OPEL), defined by having at least one parent who lived to age 95 or older, and the remaining were offspring of parents with usual survival (OPUS), defined by having neither parent survive to age 95. Demographic and clinical baseline characteristics are summarized in Table 1. The mean age of the participants at enrollment was 75.8 ± 6.7 years (age range: 65–95 years) and 55.7% of participants were women. The mean ages of male and female participants were 76.0 ± 6.8 and 75.6 ± 6.7 years, respectively.

**Table 1:** Cohort characteristics

Variables	LonGenity	OPEL	OPUS
Participants, *n* (%)	1,025	506 (49.4)	519 (50.6)
Age, mean ± SD, years	75.8 ± 6.7	74.5 ± 6.1	77.1 ± 7.1
Women, *n* (%)	571 (55.7)	306 (60.5)	265 (51.1)
Education, mean ± SD, years	17.46 ± 2.94	17.67 ± 2.95	17.24 ± 2.91
Co‐morbid conditions			
Stroke, %	3.3	1.4	5.2
Diabetes, %	8.9	6.9	10.8
Myocardial infarction, %	5.3	4.3	6.2
Hypertension, %	43.2	35.9	50.4
Rockwood frailty index (mean ± SD)	0.163 ± 0.086	0.151 ± 0.079	0.175 ± 0.091

### 2.2 Association analysis with chronological age

**Figure 1:**
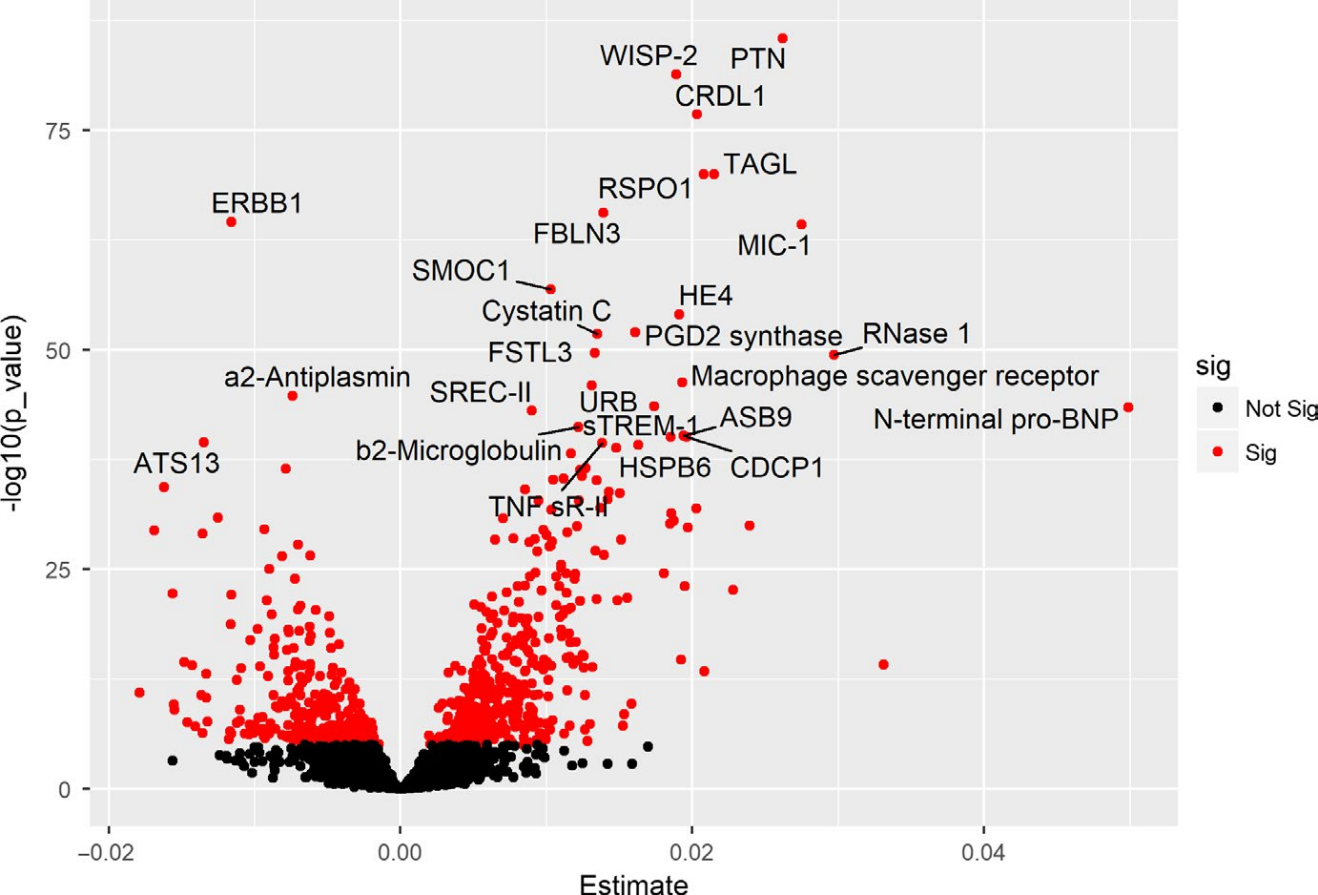
Association of proteins with chronological age. Volcano plot showing associated proteins as red dots (*p*‐value < 1.0 × 10^−5^). *x*‐axis denotes the beta estimate coefficients, and *y*‐axis, the significance level presented as −log10 (*p*‐value) from linear model adjusted for sex and cohort status. Top most hit proteins have been marked

Chronological age was significantly associated with 754 proteins (*p* < 1 × 10^−5^). Of these, the majority, 427 (56.6%) proteins, were positively associated with aging while the remaining 327 were negatively associated. The top proteins that were significantly positively correlated with age included pleiotrophin (PTN), WNT1‐inducible‐signaling pathway protein 2 (WISP‐2), chordin‐like protein 1 (CRDL1), R‐spondin‐1 (RSPO1), transgelin (TAGL), EGF‐containing fibulin‐like extracellular matrix protein 1 (FBLN3), and growth/differentiation factor 15 (MIC‐1; Table 2; Figure 1). On the other hand, epidermal growth factor receptor (ERBB1), a2‐antiplasmin, and A disintegrin and metalloproteinase with thrombospondin motifs 13 (ATS13), among others, were negatively associated with age (Table 2; Figure 1). Furthermore, we reaffirmed the associations between age and a number of proteins that had been previously found to correlated with age by other studies, including MIC‐1(GDF15), cystatin C, a2‐antiplasmin, N‐terminal pro‐BNP, b2‐microglobulin, growth hormone receptor, and IGFBP‐2 (Figure 1). Interestingly, the top most proteins associated with chronological age were also associated with age‐related complex traits such as diabetes, myocardial infarction, stroke, hypertension, gait velocity, grip strength, and frailty (Figure S1).

**Table 2:** Top 20 most significant SOMAmer reagents associated with chronological age in 1,025 participants

Target	Target full name	UniProt	Estimate	SE	*p*‐value
PTN	Pleiotrophin	P21246	0.0262	0.0012	3.21E−86
WISP‐2	WNT1‐inducible‐signaling pathway protein 2	O76076	0.0189	0.0009	4.60E−82
CRDL1	Chordin‐like protein 1	Q9BU40	0.0203	0.0010	1.45E−77
TAGL	Transgelin	Q01995	0.0215	0.0011	9.70E−71
RSPO1	R‐spondin‐1	Q2MKA7	0.0208	0.0011	1.09E−70
FBLN3	EGF‐containing fibulin‐like extracellular matrix protein 1	Q12805	0.0139	0.0007	2.62E−66
ERBB1	Epidermal growth factor receptor	P00533	−0.0116	0.0006	2.87E−65
MIC‐1	Growth/differentiation factor 15	Q99988	0.0275	0.0015	5.15E−65
SMOC1	SPARC‐related modular calcium‐binding protein 1	Q9H4F8	0.0103	0.0006	1.23E−57
HE4	WAP four‐disulfide core domain protein 2	Q14508	0.0191	0.0012	9.27E−55
PGD2 synthase	Prostaglandin‐H2 D‐isomerase	P41222	0.0161	0.0010	9.42E−53
Cystatin C	Cystatin C	P01034	0.0135	0.0008	1.65E−52
FSTL3	Follistatin‐related protein 3	O95633	0.0133	0.0008	2.49E−50
RNase 1	Ribonuclease pancreatic	P07998	0.0297	0.0019	3.66E−50
Macrophage scavenger receptor	Macrophage scavenger receptor types I and II	P21757	0.0193	0.0013	4.77E−47
URB	Coiled‐coil domain‐containing protein 80	Q76M96	0.0131	0.0009	1.14E−46
a2‐Antiplasmin	Alpha‐2‐antiplasmin	P08697	−0.0074	0.0005	1.77E−45
sTREM‐1	Triggering receptor expressed on myeloid cells 1	Q9NP99	0.0174	0.0012	2.54E−44
N‐terminal pro‐BNP	N‐terminal pro‐BNP	P16860	0.0499	0.0034	3.73E−44
SREC‐II	Scavenger receptor class F member 2	Q96GP6	0.0090	0.0006	8.37E−44

Model: log(SOMAmer) ~ age + gender + cohort.

#### 2.2.1 Association analysis stratified by cohort status and sex

**Figure 2:**
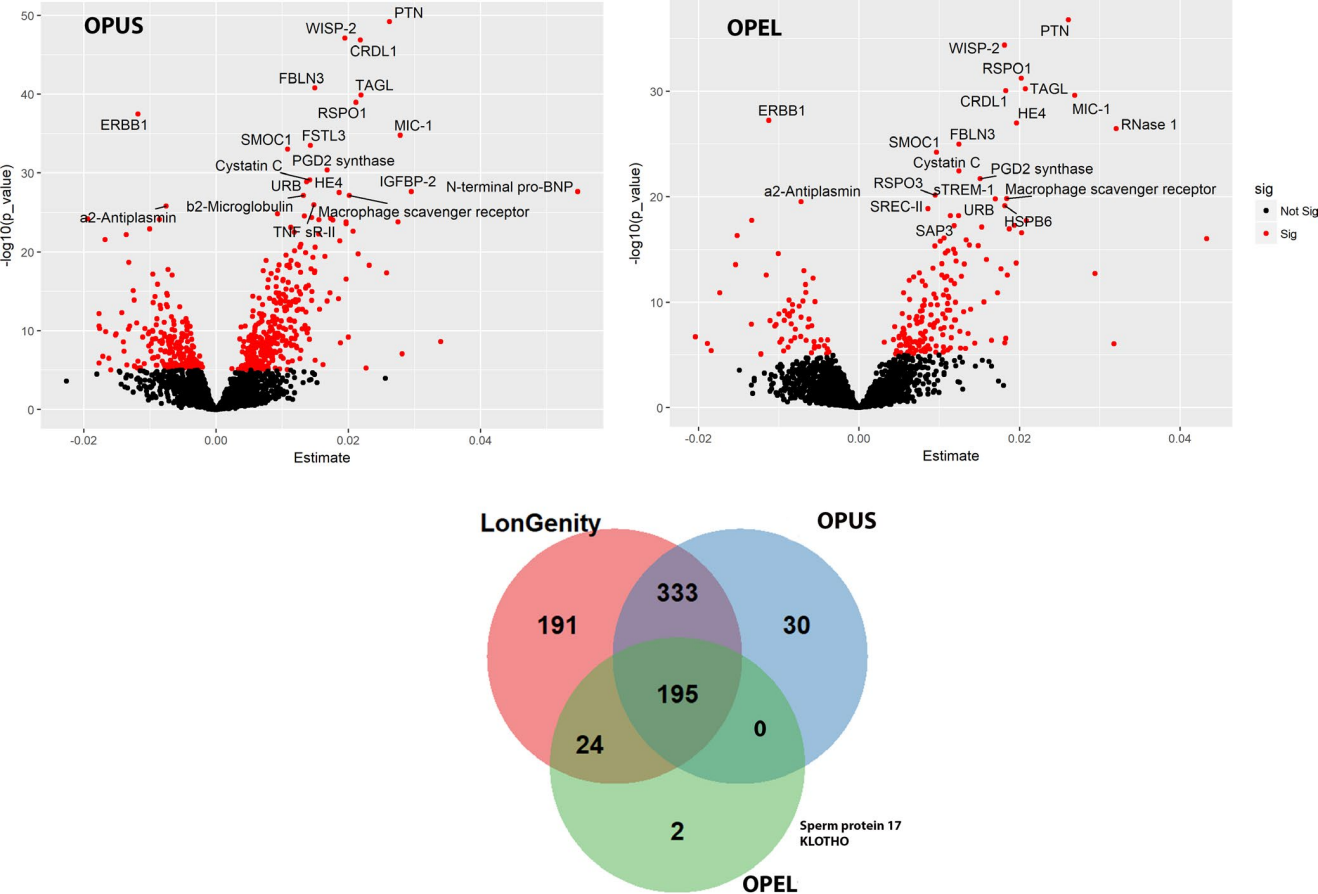
Association of proteins with chronological age in OPEL and OPUS: (Panel a) Volcano plot showing associated proteins as red dots (*p*‐value < 1.0 × 10^−5^). *x*‐axis denotes the beta estimate coefficients from linear model, and *y*‐axis shows the significance level presented as −log10 (*p*‐value). Top most hit proteins have been marked. (Panel b) Venn diagram showing overlap between associated proteins in entire cohort (LonGenity), OPEL and OPUS

In an analysis stratified by cohort status, we identified 228 proteins significantly associated with age in OPEL and 568 proteins associated with age in OPUS. While most of these age‐associated proteins were common to OPEL and OPUS (Figure 2; Tables S1 and S2), 26 proteins were reproduced only among OPEL in the stratified analysis while two proteins, KLOTHO and sperm protein 17, were completely unique to OPEL and only emerged as significant after stratification (Figure 2).

**Figure 3:**
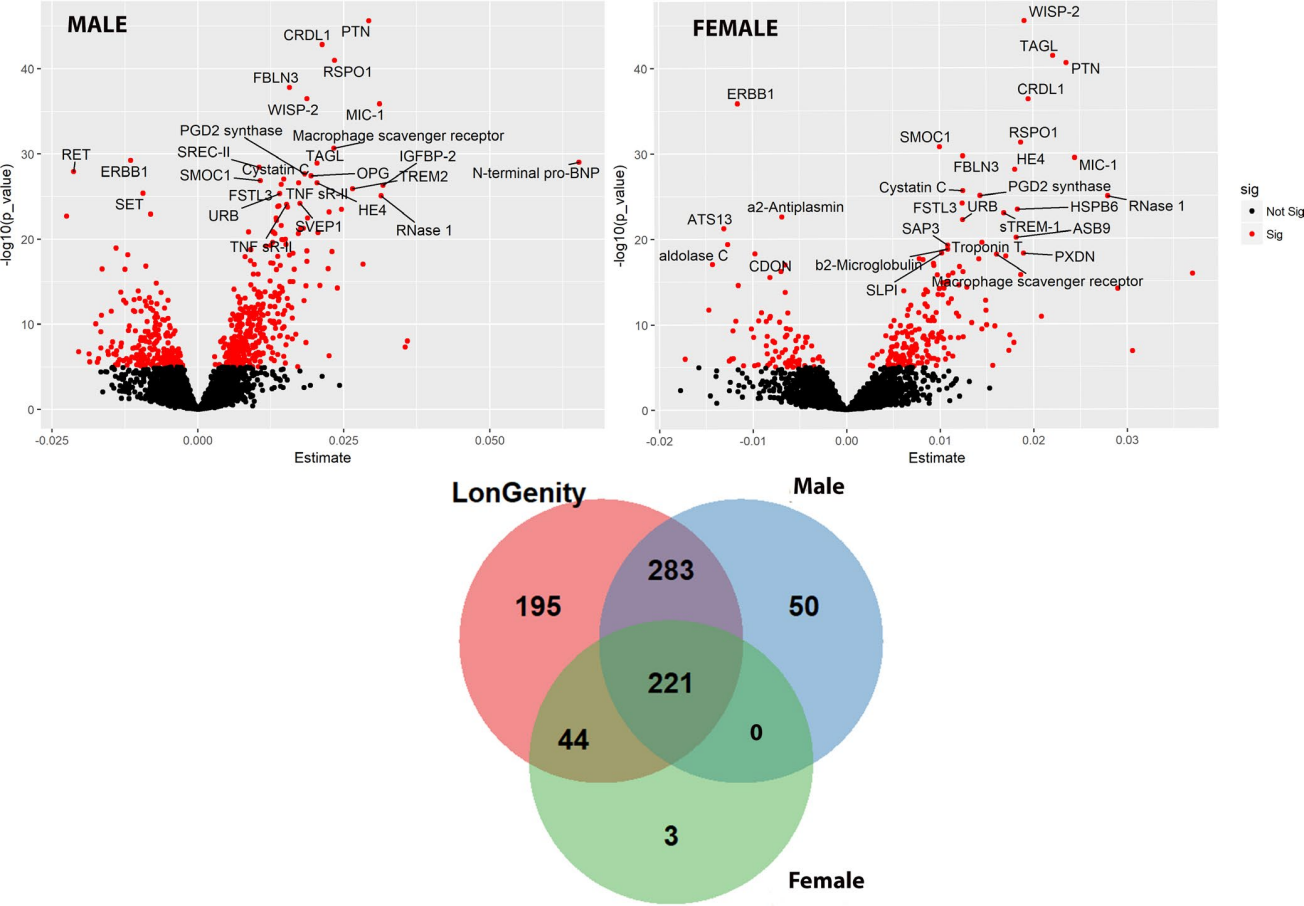
Association of proteins with Chronological age in Males and Females: (Top) Volcano plot showing associated proteins as red dots (*p*‐value < 1.0 × 10^−5^). *x*‐axis denotes the beta estimate coefficients from linear model, and *y*‐axis shows the significance level presented as −log_10_ (*p*‐value). Top most hit proteins have been marked. (Below) Venn diagram showing overlap between associated proteins in the entire cohort and included males and females

In a sex‐stratified analysis, there were 564 significant age‐associated proteins in males compared to 274 proteins in females. In both sexes, 221 proteins were common (Figure 3). However, while PTN was most strongly associated with age among males (Table S3), WISP‐2 was the top protein associated with age in females (Table S4).

Reactome pathway analysis found that “insulin‐like growth factor (IGF) transport and regulation” pathway was most strongly associated with age, followed by pathways involved in extracellular matrix remodeling, post‐translational modification and clotting (Table S5). Analysis using IPA identified pathways related to cell growth, development, and survival, and inflammatory response, cancer, and cardiovascular diseases to be the top pathways related to aging (Table S6).

### 2.3 Co‐expression network analysis and phenotypic association

**Figure 4:**
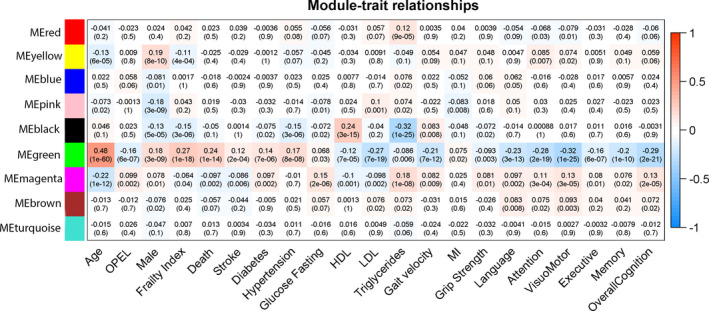
Module‐trait associations: Each row corresponds to a module eigengene, column to a trait. Each cell contains the corresponding correlation and *p*‐value. The table is color‐coded by correlation according to the color legend. Green module was associated with age and diverse traits. Magenta module was second top hit with age in inverse direction

Next, we performed an unbiased weighted gene co‐expression network analysis (WGCNA) in order to investigate the association between protein networks and age. In this analysis, 4,265 proteins were clustered into nine modules based on co‐expression analysis done in our subjects, with each module characterized by module eigengene (ME). The nine modules include black (230 proteins), blue (1,026 proteins), brown (486 proteins), green (420 proteins), magenta (31 proteins), pink (98 proteins), red (390 proteins), turquoise (1,126 proteins), and yellow (458 proteins) (Figures 4 and S2).

Chronological age was most significantly associated with the green module (Cor = 0.48, *p* = 1.0 × 10^−60^). Gene significance (GS) for age and module membership in green module showed significant correlation (Cor = 0.69, *p* = 1.2 × 10^−60^; Figure S3). Top hub gene in this module was tumor necrosis factor receptor 1 (TNFR1). The pathways enriched in this module included inflammatory response, ECM remodeling, IGF transport, and complement cascade (Table S7).

ME green was also associated with aging‐related complex traits such as stroke (Cor = 0.12, *p* = 2 × 10^−4^), diabetes (Cor = 0.14, *p* = 7 × 10^−6^), hypertension (Cor = 0.17, *p* = 8 × 10^−8^), frailty (Cor = 0.27, *p* = 1 × 10^−18^), and mortality (Cor = 0.24, *p* = 1 × 10^−14^). This module was also negatively associated with gait velocity (Cor = −0.21, *p* = 7 × 10^−12^) and overall cognition (Cor = −0.29, *p* = 2 × 10^−21^; Figure 4). Interestingly, this module was negatively correlated with OPEL status (Cor = −0.16, *p* = 6 × 10^−7^) and positively associated with male gender (Cor = 0.18, *p* = 3 × 10^−9^) (Figure 4).

The ME Magenta module demonstrated associations with age‐related traits that diverged from those of the ME green. The Magenta module was negatively associated with age (Cor = −0.22, *p* = 1 × 10^−12^) and nominally with less frailty, death, and stroke (Figure 4). This module was also positively associated with higher cognitive scores and physical measures such as grip strength and gait velocity. The pathways that made up the Magenta module included those related to metabolism–energy production, lipid metabolism, endocrine, and digestive system development and function (Table S8). The top hub gene in this module was fructose‐1, 6‐bisphosphatase 1 (F16P1).

### 2.4 Age prediction

**Figure 5:**
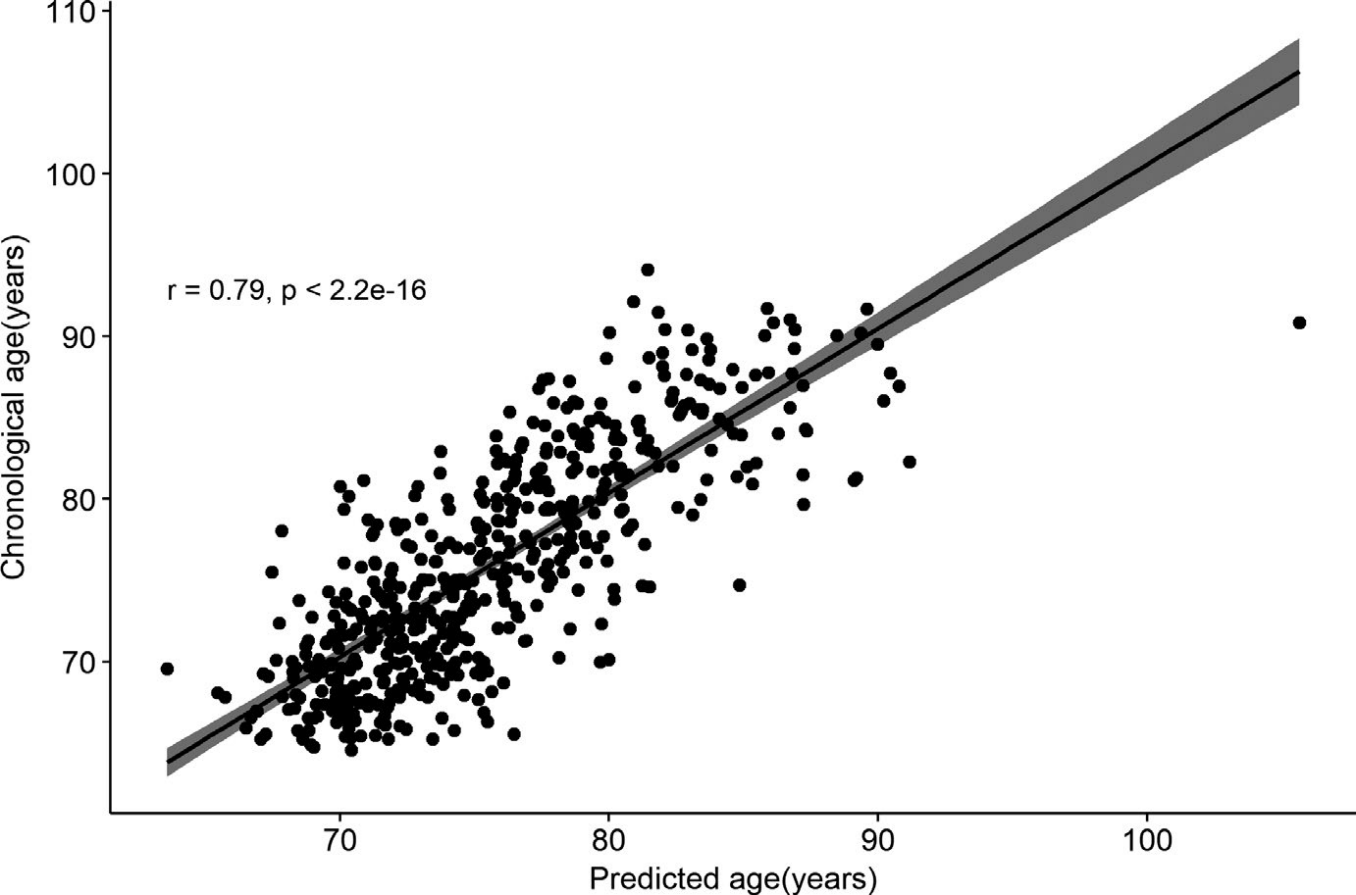
Correlation of chronological age and predicted age using proteomic data: Age prediction was carried out using elastic net regression method in 525 participants in the validation set. Correlation of predicted age using proteomic markers and chronological age was ~0.8

An elastic net regression model which aimed to select proteins that predicted chronological age identified 162 relevant proteins from 4,265 proteins and 61 of those proteins 162 proteins were associated with chronological age (Table S9). The correlation between chronological age and the age predicted by our model (proteomic age) was *r* = 0.79 (*p* < 2.2E−16; Figure 5). Alternatively, we successfully created age predictor from top significant 200, 100, and 50 proteins associated with chronological age. 74, 67, and 35 proteins, respectively, were selected in elastic net regression model out of 200, 100, and 50 proteins (Tables S10–S12). The correlation with chronological age and the age predicted were comparable to primary model with correlation of 0.79 (*p* < 2.2E−16), 0.80 (*p* < 2.2E−16), and 0.78 (*p* < 2.2E−16), respectively (Figure S4).

**Figure 6:**
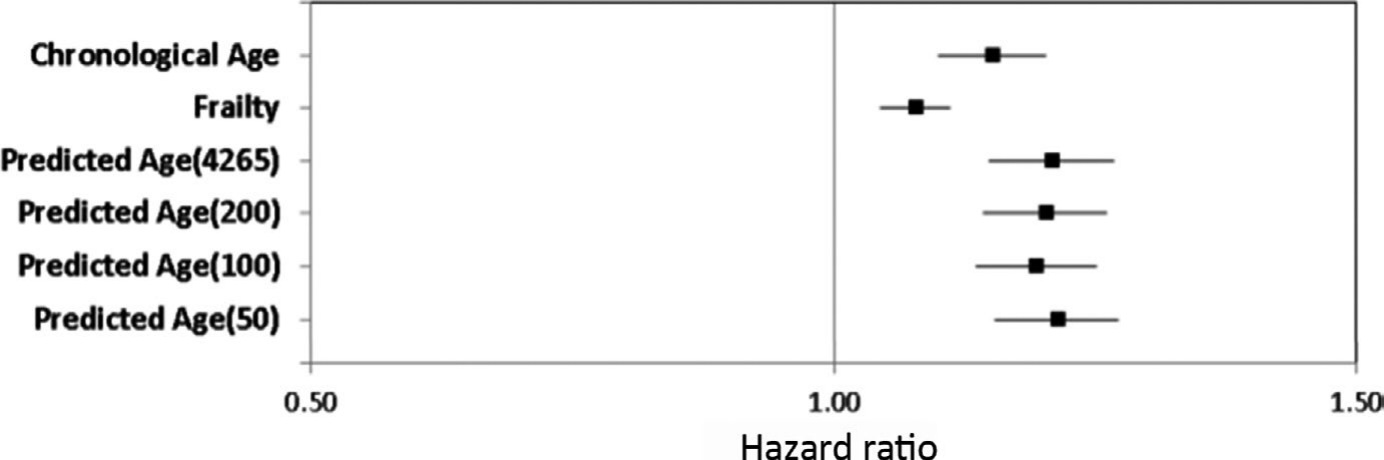
Prediction of all‐cause mortality by chronological age, cumulative frailty index, and proteomic age derived using different methods (the Cox regression analysis). Analysis was adjusted for gender and cohort. *162, 74, 67, and 35 proteins were selected using elastic net regression for prediction from 4,265 (total proteins), top 200, 100, and 50 age‐associated proteins, respectively

We compared the predictive validity of chronological age, constructed proteomic age, and the frailty index for all‐cause mortality. The median follow‐up time was 4.79 ± 2.88, and 51 deaths were reported in 525 participants in the validation set. The proteomic age predicted all‐cause mortality better than chronological age (HR 1.21, 95% CI 1.15–1.27, *p* = 3.10E−14 vs. HR 1.15, 95% CI 1.10–1.20, *p* = 2.54E−10, respectively) and cumulative frailty index (HR 1.08, 95% CI 1.05–1.11, *p* = 9.62E−07; Figure 6). All our secondary prediction models too showed similar result with mortality (Figure 6). When all three predictors were included in a single model, proteomic age predicted all‐cause mortality (HR 1.12, 95% CI 1.04–1.21, *p* = 0.004) better than chronological age (HR 1.07, 95% CI 1.00–1.14, *p* = 0.04) and the frailty index (HR 1.03, 95% CI 0.99–1.06, *p* = 0.08) in the unified model.

The extended results for association analysis with chronological age and module classification are provided in Tables S13–S18.

## 3 DISCUSSION

The present study identified proteomic profiles associated with chronological age and proteomic signatures related to aging phenotypes in a unique population of older adults (Ayers et al., 2014; Gubbi et al., 2017). Maintenance of homeostasis is important in successful aging, whereas major deviations from stable physiology that can be captured by changes in the proteome may reflect accelerated aging and disease prevalence (Basisty et al., 2018). Our findings demonstrated that individuals with a family history of longevity exhibit a proteome that is suggestive of delayed aging. Additionally, by utilizing the WGCNA approach we showed that clusters of proteins, which were associated with age, were also related to complex diseases and other age‐associated phenotypes. These findings support prior research, which demonstrated that age is a common risk factor for most aging‐associated complex diseases, syndromes, and traits (Kaeberlein et al., 2015). Moreover, the proteomic age model developed in the present study predicted mortality more precisely than chronological age and frailty.

Our study was conducted in one of the largest older cohorts of older adults who were phenotyped with a proteomic panel consisting of 4,265 SOMAmer reagents (Menni et al., 2014; Tanaka et al., 2018). In addition to confirming prior findings that associated observations of MIC‐1, PTN, CRDL1, and N‐terminal pro‐BNP associations with age (Menni et al., 2014; Tanaka et al., 2018), we identified new proteins and proteomic profiles correlated with aging. The study carried out in BLSA and GESTALT cohorts identified 217 of 1,301 proteins to be associated with age (Tanaka et al., 2018). The present study found 754 of 4,265 proteins to be correlated with chronological age. Interestingly, in comparison with other studies, we identified many more proteins that were down‐regulated with age (<10% vs. 43%, respectively; Tanaka et al., 2018). Of note, the small fraction of down‐regulated proteins reported by Toshiko et al. was much less than reported by other studies that used alternate methodologies for proteomic analysis and found equilibrium between the number of up‐regulated and down‐regulated proteins (Tanaka et al., 2018; Waldera‐Lupa et al., 2014). Thus, it is likely that the expanded version of the SomaScan assay utilized here was more inclusive and reflected the proteomic changes that accompany aging in humans more comprehensively.

*Pleiotrophin (PTN)*, also known as heparin binding growth factor, was the protein most strongly associated with chronological age in our study. PTN is secreted as a cell signaling cytokine, and as its name suggests, it is involved in a plethora of functions such as cell growth, migration, and survival in diverse tissues, including brain and bone. In the central nervous system, PTN acts as an important neuromodulator that plays a role in neurogenesis, learning, and long‐term memory (González‐Castillo et al., 2015). In the bone, PTN is known as OSF‐1, and it is involved in bone formation and repair, and osteoprogenitor differentiation and proliferation (Lamprou et al., 2014). These pleiotropic functions result from PTN’s ability to bind to different receptors, including receptor protein tyrosine phosphatase ζ (RPTPζ), anaplastic lymphoma kinase (ALK), neuroglycan C, N‐syndecan receptor, and low‐density lipoprotein receptor‐related protein (LRP; González‐Castillo et al., 2015). The functionality and mechanism of action of PTN are not completely elucidated.

Another protein associated with age in our analysis, *WNT1*‐*inducible*‐*signaling pathway protein 2 (WISP*‐*2)*, may have pro‐survival effects by contributing to Wnt3a mediated vascular smooth muscle survival (Brown et al., 2019). It may have a particular role in protection from atherosclerosis, as WISP‐2 was shown to have anti‐fibrotic effects that protect from cardiac hypertrophy and fibrosis (Grünberg et al., 2018). WISP‐2 was also shown to promote mesenchymal precursor cell growth and to have pro‐survival role in IGF‐1 stimulated islet cell growth and survival (Chowdhury et al., 2014). Its potential ability to preserve tissue growth and survival suggests that WISP‐2 may play an important role in longevity. Other top proteins associated with age include *chordin*‐*like 1 (CRDL1)*, a bone morphogenetic protein‐4 antagonist, that also has been identified in other studies employing the SomaScan platform (Menni et al., 2014) and *R*‐*spondin*‐*1*, a Wnt agonist that amplifies Wnt signaling. Studies have shown that reduced exposure to R‐spondin‐1 partially rescues stem cell differentiation in old mice (Cui et al., 2019).

The top proteins identified in relationship with age point toward potential novel aging mechanisms and pathways. In addition, this study found proteins that had been previously associated with both diseases and age, including growth differentiation factor‐15 (GDF‐15) and NT‐pro‐BNP. The relationship between GDF‐15 and age has been noted in a recent SomaScan study, as has been its association with diabetes, cardiovascular disease, and mortality (Tanaka et al., 2018). NT‐pro‐BNP is a known risk factor for coronary artery disease and is associated with mortality in patients with heart disease (Kragelund et al., 2005). Furthermore, our analysis confirmed associations of top age‐related proteins with complex diseases and traits such as diabetes, myocardial infarction, stroke, hypertension, gait velocity, grip strength, and frailty. The overlap of age‐associated proteins with aging‐related diseases and syndromes signals to common mechanisms that potentially can be targeted by drugs such as metformin (Barzilai et al., 2016).

We identified more age‐associated proteins in men compared to women and in OPUS compared to OPEL. Worldwide, women have longer life spans than men; however, the underlying cause of this difference has not been delineated (Austad, 2006). Proposed theories to explain the life‐span difference include the effect of estrogen, additional X chromosome advantage, and gender specific effect of growth hormone/IGF‐1(Ashpole et al., 2017; Christensen et al., 2001). Additionally, there are reports of higher incidence of DNA damage /mutations in males compared to females (Fischer & Riddle, 2017). The noted difference in the number of age‐related proteins between men and women in this study also suggests that compared to males, females may maintain better proteostasis function. A similar relationship between age and proteins was observed in OPUS compared to OPEL. Furthermore, OPEL demonstrates better health and physical characteristics compared to OPUS (Ayers et al., 2014; Gubbi et al., 2017). Interestingly, age‐associated expression of KLOTHO protein was noted only in OPEL. Our earlier study had shown differential distribution of KLOTHO genotypes with age (Bergman et al., 2007).

Age is a risk factor for a wide range of complex diseases and traits (Kaeberlein et al., 2015). Our analysis suggested a shared etiology between multiple phenotypes, with age acting as the common risk factor. For example, the Green module that was most strongly associated with age was also associated with age‐related diseases and phenotypes, such as frailty, gait velocity, and cognition. Interestingly, the Green module was negatively associated with OPEL status, who have been shown to age more successfully than OPUS (Ayers et al., 2014). This module was enriched with inflammatory response proteins, as well as those associated with cell death and survival. Inflammation plays an important role in aging and in complex disease pathogenesis (Furman et al., 2019). The top hub protein in the green module, tumor necrosis factor receptor superfamily member 1A (TNF sR‐I), is a receptor for TNF‐α and is involved in inflammation, apoptosis, and cell survival (Parameswaran & Patial, 2010). On the other hand, the Magenta module was negatively associated with age, as well as with most age‐related diseases and phenotypes, with the exception of serum glucose and diabetes. Previous studies have suggested possibility of glucose intolerance to be protective for aging (Barzilai & Ferrucci, 2012). The Magenta module is enriched with proteins that are part of the energy metabolism pathways, which have been shown to play an important role in longevity (Barzilai et al., 2012). The top hub protein in this module was Fructose 1, 6 bisphosphatase 1 (FBPase1), a rate‐limiting enzyme in gluconeogenesis. Gluconeogenesis has been shown to be enhanced with aging and attenuation of gluconeogenesis is known to extend the cellular life span (Hachinohe et al., 2013). These observations again highlight an important concept that targeting aging, the common cause of multiple diseases, rather than each disease individually may be a preferred approach for extending human health span.

Pathway analysis involving age‐associated proteins showed *regulation of insulin*‐*like growth factor (IGF) transport and uptake by insulin*‐*like growth factor*‐*binding proteins (IGFBPs)* (R‐HSA‐381426) to be the top pathway related to age. IGF‐1 is an endocrine and autocrine/paracrine growth factor that has diverse effects on development, cell growth, differentiation, and tissue repair (Higashi et al., 2012). IGF‐1 signaling pathway has been implicated in longevity and in age‐related diseases (Rincon et al., 2005). Other pathways significantly associated with age, including *degradation of the extracellular matrix* (R‐HSA‐1474228), *post*‐*translational protein phosphorylation* (R‐HSA‐8957275), *platelet degranulation* (R‐HSA‐114608), and *formation of fibrin clot* (Clotting Cascade; R‐HSA‐140877) may also have important implications for functional aging, as they have been shown to be involved in processes such as maintenance of skeletal muscle integrity, Alzheimer's disease, platelet function, and extracellular matrix degradation (Jacob, 2003; Martin et al., 2011).

The strengths of our study include a well‐characterized longitudinal homogenous cohort and the largest panel of proteins targeted using the SomaScan assay reported to date. However, there are a number of limitations in respect to the evolving SomaScan technology. This technology captures proteins based on the 3D protein structure using aptamers that bind to specific binding sites on the proteins. Therefore, there is a possibility that a protein that has a change in this binding site may be missed or that the aptamer may cross‐react with another protein that has a similar binding site. Additionally, this technology does not measure the absolute concentration of proteins, but expresses the concentration as an amount of SOMAmer reagent captured. This precludes direct correlations with results derived by other methods. Moreover, we found that ~17% of proteins associated with age in our study that used 4,265 SOMAmer reagents, a similar percentage compared to another study that included 1,301 SOMAmers (Tanaka et al., 2018). If indeed 17% of the entire human proteome is associated with aging, then we may be missing an important component of the aging proteome, with the remaining proteins yet to be discovered in a pool of more than 20,000 proteins and their isoforms. In addition, although the present study found a unique repertoire of proteins to be associated with aging and have shown predicted age to be better marker than chronological age for mortality, the results are yet to be replicated in independent cohorts.

In conclusion, we identified a number of proteins and pathways significantly associated with chronological age in a population of older adult and showed that proteomic profiles can be better predictors of biological age—mortality and disease—than chronological age. These discoveries pave the way for better risk stratification for older adults and the identification of novel pathways that modulate aging, which can be targeted with the goal of delaying aging and age‐related diseases.

## 4 METHODS

### 4.1 LonGenity cohort

The LonGenity study is an ongoing longitudinal study established in 2007 that recruits Ashkenazi Jewish participants age 65 and older. The cohort consist of adults who were either offspring of parents with exceptional longevity (OPEL, defined by having at least one parent who lived to age 95 or older) or offspring of parents with usual survival (OPUS, defined by having neither parent survive to age 95). The primary goal of this longitudinal study was to identify genotypes that confer longevity and successful aging. Participants were recruited systematically using public records such as voter registration lists or through contacts at community organizations, synagogues, and advertisements in Jewish newspapers in the New York City area. Potential participants were contacted by telephone to assess interest and eligibility. Exclusion criteria include the following: a score >2 on the AD8 (Galvin et al., 2005) and >8 on the Blessed Information–Memory–Concentration task (Blessed et al., 1968) at the initial screening interview, having a sibling in the study, and severe visual impairment. Participants who were eligible were invited to our research center for further evaluation. Participants received detailed medical history evaluation, functional evaluation, and cognitive testing at baseline and at annual follow‐up visits. As part of their annual visit, participants completed neuropsychological tests evaluating memory, language, visuospatial functioning, attention, and executive function under the supervision of the study neuropsychologist. An overall cognition composite score was calculated by transforming participant scores into a standard score adjusted for education, age and gender and summing the standard scores.

All participants signed written informed consents for study assessment and genetic testing prior to enrollment. The Albert Einstein College of Medicine Institutional Review Board approved the study protocol.

### 4.2 Proteomic assessment

Proteomic assessment was carried out in LonGenity cohort using SomaScan assay. Plasma was isolated from EDTA‐treated blood acquired by venipuncture from participants at baseline wave in a fasting state. Plasma samples were stored at −80°C, and 150 µl of aliquots of plasma was sent to SomaLogic on dry ice. This study used 5 k SomaScan Assay V4 which had 5284 SOMAmer reagents, with 5209 SOMAmer reagents targeting human proteins and remaining markers consisting of 22 non‐human proteins, 12 hybridization control elution, 10 non‐biotin, four non‐cleavable, and seven deprecated proteins, and 20 spuriomers. SomaScan data standardization was carried out as previously described (Candia et al., 2017) at SomaLogic, Inc. Three main steps included hybridization control normalization (HCN), median signal normalization (MSN), and calibration normalization (CN). HCN removed individual sample variance on the basis of signaling differences between microarrays or Agilent scanners while MSN removed inter‐sample variation within a plate for calibrator and buffer samples arising from pipetting variation or other technical issues. CN removed variance across assay runs. Finally, median normalization to reference was performed on the quality control (QC) and individual samples to control for inter‐sample technical and biological variability in total signal within and between runs. After implementing these QC checks, 960 sequences that failed QC were removed. After exclusion of non‐human proteins, deprecated markers, non‐cleavable, non‐biotin, and spuriomers, 4265 SOMAmer reagents were available for the proteomic analysis.

### 4.3 Statistical analysis

Baseline characteristics of participants were compared using descriptive statistics. Relative fluorescence unit (RFU) values observed after data normalization procedure for each SOMAmer reagent were natural log‐transformed. Outliers were removed using median absolute deviation method. The preliminary objective of this study was to identify the association of SOMAmer reagents with chronological age using linear regression analysis. Analyses were adjusted for gender and cohort status (OPUS or OPEL). Beta estimate is defined as increase or decrease in specific log (SOMAmer reagent) concentrations with each 1 unit (1 year) of increase in age. Initial normalization procedures carried out by SomaLogic adjusted for changes associated with the experimental setup like inter‐sample differences within a plate and variance across assay runs, individual sample variance on the basis of signaling differences between microarrays or Agilent scanners. The Bonferroni corrected *p*‐value less than 1.0 × 10^−5^ (0.05/4,265) were considered significant. Gender and cohort stratified analyses were performed to understand the possible differential effect of gender and cohort status on age regulated proteomic profile.

### 4.4 Pathway analysis

Pathway or enrichment analyses were carried out using proteins associated with chronological age to discover biological pathways related to aging. Network analysis was carried out using Qiagen's Ingenuity^®^ Pathway Analysis (IPA^®^, QIAGEN Redwood City, www.qiagen.com/ingenuity; Krämer et al., 2013). For this analysis, we included 754 proteins that were significantly associated with chronological age in our analysis. IPA *network analysis* output consisted of a list of biological functions and set of proteins, as well as a score (*p*‐score = −log10 (*p*‐value)) according to the fit of the protein set. We also investigated *top diseases and bio*‐*functions* associated with aging. Top networks were checked for concordance with pathway analysis using Reactome (www.reactome.org/; Fabregat et al., 2017). The database was queried with the UniProt IDs to check whether particular pathways were over‐represented.

### 4.5 Weighted gene co‐expression network analysis

The WGCNA R package (Langfelder & Horvath, 2008) was used to build unsigned protein expression networks from normalized and transformed RFUs of 4265 SOMAmers concentrations. The WGCNA methodology has been well described in previous publications and the tutorial accompanying this R package (Langfelder & Horvath, 2008). In our dataset, the smallest threshold satisfying scale free topology fit of *R* ^2^ = 0.90 was found at soft threshold power of 2. Topological overlap matrix (TOM) is used to express network interconnectedness. Hierarchical clustering of proteins were based on topology overlap dissimilarity (1‐TOM), and modules were defined from branches of cluster trees using dynamic tree cut method (Langfelder et al., 2007). Modules were assigned with different color names. Minimum module size was set at default of 30 proteins.

First principle component of a module is defined as Module eigengene *E*. This is used as representative measure of module expression profile. Further association of the module to the phenotype of interest was carried out by correlating module eigengene with the outcome phenotype. We analyzed the association of the module with the primary phenotype of interest (chronological age) and other age‐associated phenotypes including frailty index, death, stroke, diabetes, hypertension, myocardial infarction, lipid levels, physical measures (grip strength, and gait velocity), and cognitive phenotypes (language, attention, executive, memory, and visuomotor). We have selected all prominent age‐associated phenotypes whose data were available in our cohort.

Each module is characterized by a highly connected gene called a hub gene. A hub gene was defined based on highest module membership (MM). MM is measured as correlation of individual protein expression profile with the module eigengene of a given module. Hub genes were analyzed for associated module with age.

### 4.6 Proteomic prediction of chronological age

We constructed a proteomic chronological age predictor using penalized regression model with the glmnet R package (Friedman et al., 2009). Participants in the training set were selected using stratified random sampling method. Participants were selected from 5‐year age bins (65–70, 70–75, 75–80, 80–85, 85–90 and 90–95). The training set included 500 participants, and the remaining 525 participants of the cohort were used in a validation set. As a first step, chronological age was regressed on 4,265 log‐transformed protein abundances. Using cv‐glmnet function, optimal lambda value to minimize cross‐validation prediction error rate was selected on the basis of 10‐fold cross‐validation using the training set. Alpha value was set at 0.5 for performing elastic net regression. As a secondary analysis, we constructed prediction model including only topmost age‐associated proteins (200, 100, and 50) in the regression model. The intention of this model was to figure out possibility of modeling a clock consisting of only age‐associated proteins. A comparison was carried out with the primary model which included 4,265 proteins.

### 4.7 Survival analysis

The Cox proportional hazard models were used to compute hazard ratios (HRs) with 95% confidence intervals (CIs) to predict incident all‐cause mortality based on chronological age, proteomic age (predicted), and frailty index. We constructed cumulative frailty index in our cohort as discussed in the supplementary methods. All models were adjusted for gender and cohort status. Time scale was follow‐up time in years to date of death or final contact. Proportional hazard assumptions of all models were tested graphically and analytically and were adequately met. All survival analyses were carried out using coxph() function in R.

## CONFLICT OF INTEREST

None declared.

## AUTHOR CONTRIBUTIONS

Nir Barzilai, Joe Verghese, Sofiya Milman, and Sanish Sathyan contributed to the design of the study and interpretation of the data. Sanish Sathyan, Erica F. Weiss, Sofiya Milman, Joe Verghese, and Nir Barzilai contributed to the acquisition of data and writing of the manuscript. Sanish Sathyan, Emmeline Ayers, Tina Gao, and Nir Barzilai contributed to the analysis of the data. Sanish Sathyan, Emmeline Ayers, Tina Gao, Erica F. Weiss, Sofiya Milman, Joe Verghese, and Nir Barzilai contributed to the critical revisions of the manuscript. All the authors approved the final version of the manuscript and agreed to be accountable for all aspects of the work.

## Supporting information

Supplementary Material

Tables S9–S18

## Data Availability

Proteomic data used in this study are available upon request. Please contact the corresponding author for further information.
