# Supplementary material for: Plasma proteomic profile of age, health span, and all‐cause mortality in older adults
Source: Aging Cell. 2020 Oct 22;19(11):e13250. doi: 10.1111/acel.13250 (PMC7681045; doi:10.1111/acel.13250)
Supplement: Supplementary file 1 — Supplementary Material [file ACEL-19-e13250-s001.docx]

**Supplementary Figure 1: Age associated proteins and age associated phenotype.**

**
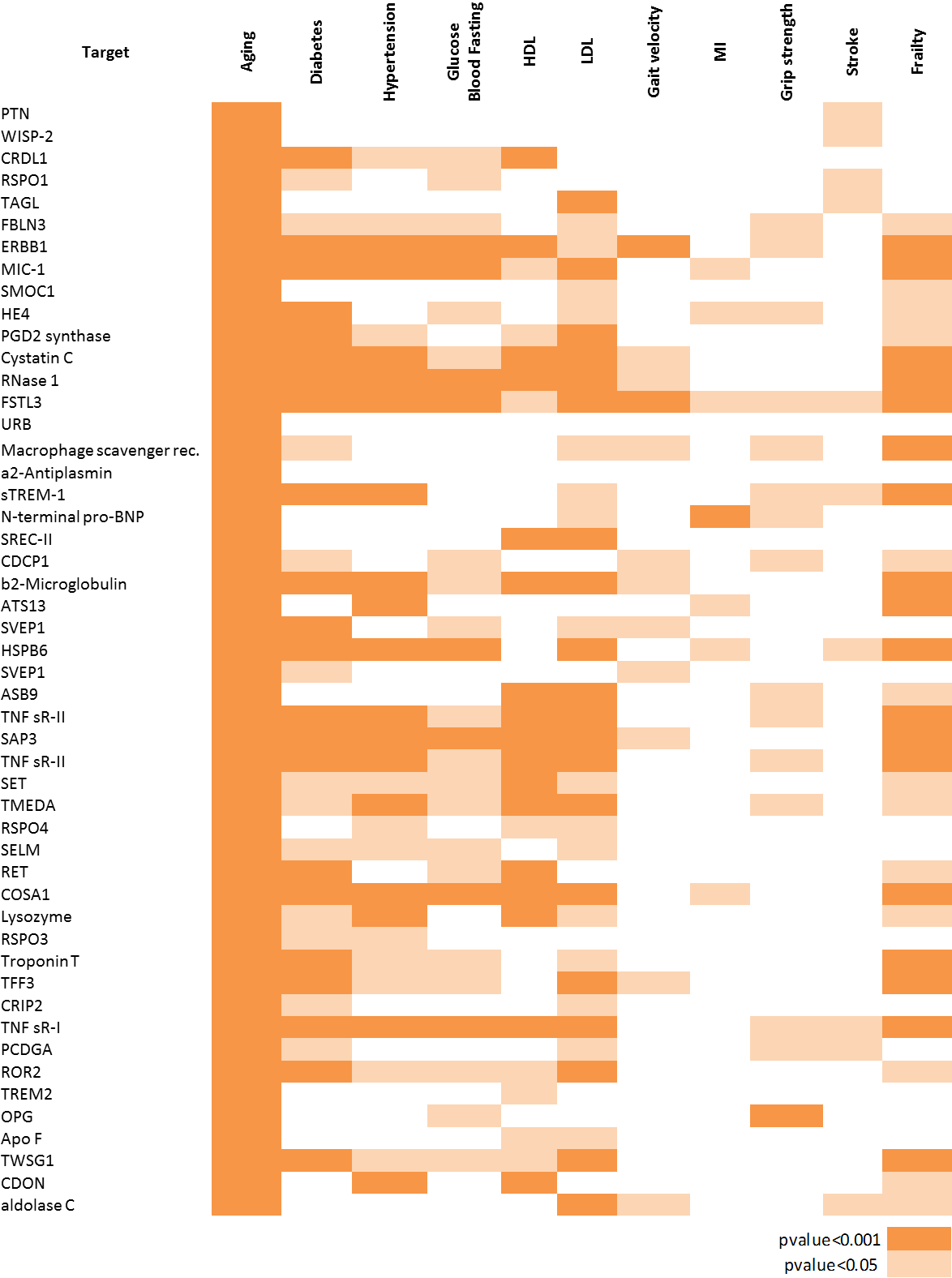
**

**Supplementary Figure2: Clustering dendrogram of 4265 proteins, with dissimilarity based on topological overlap, together with assigned module colors.** Dynamic tree cut algorithm groups genes into modules

**
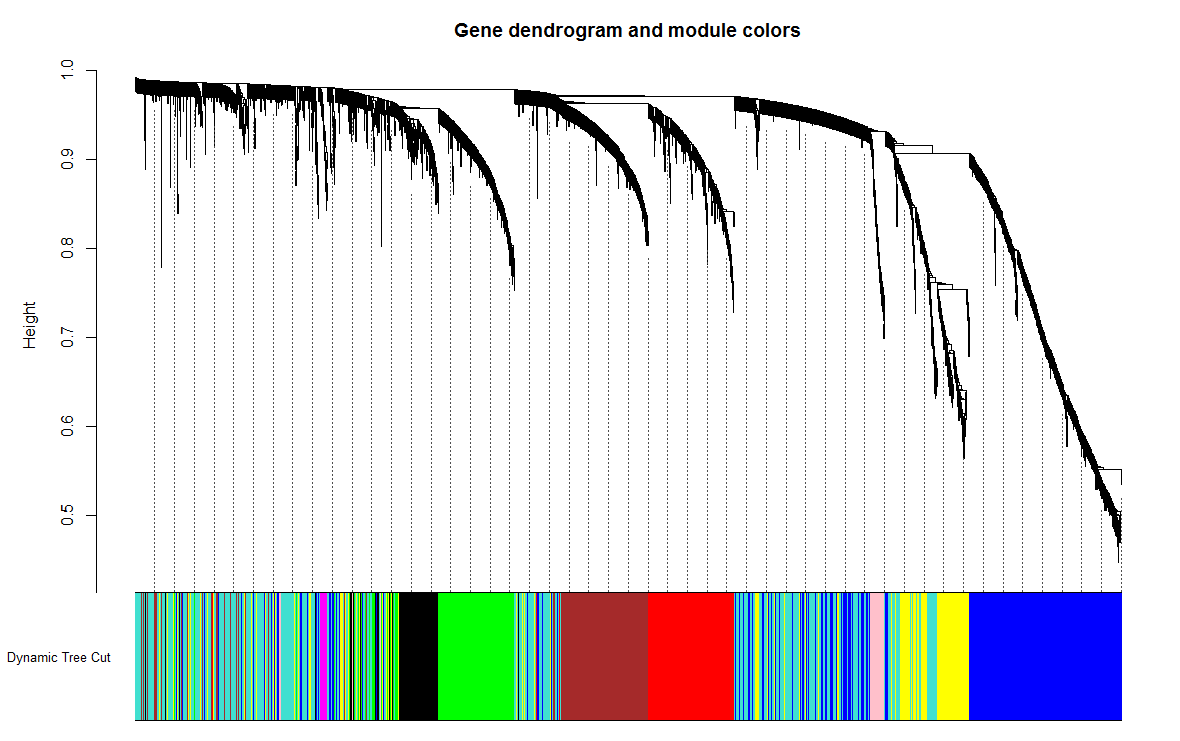
**

**Supplementary Figure 3: (a) Analysis of network topology for various soft-thresholding powers. Left:** Power 2 was lowest possible power term where topology approximately fits a scale free network (on or above red horizontal line). **Right:** mean connectivity drops as power goes up below. (b) A scatterplot of Gene Significance (GS) for Age vs. Module Membership (MM) in the green module. There is a highly significant correlation between GS and MM in this module. (c) Visualization of the eigengene network representing the relationships among the modules and the clinical trait Age


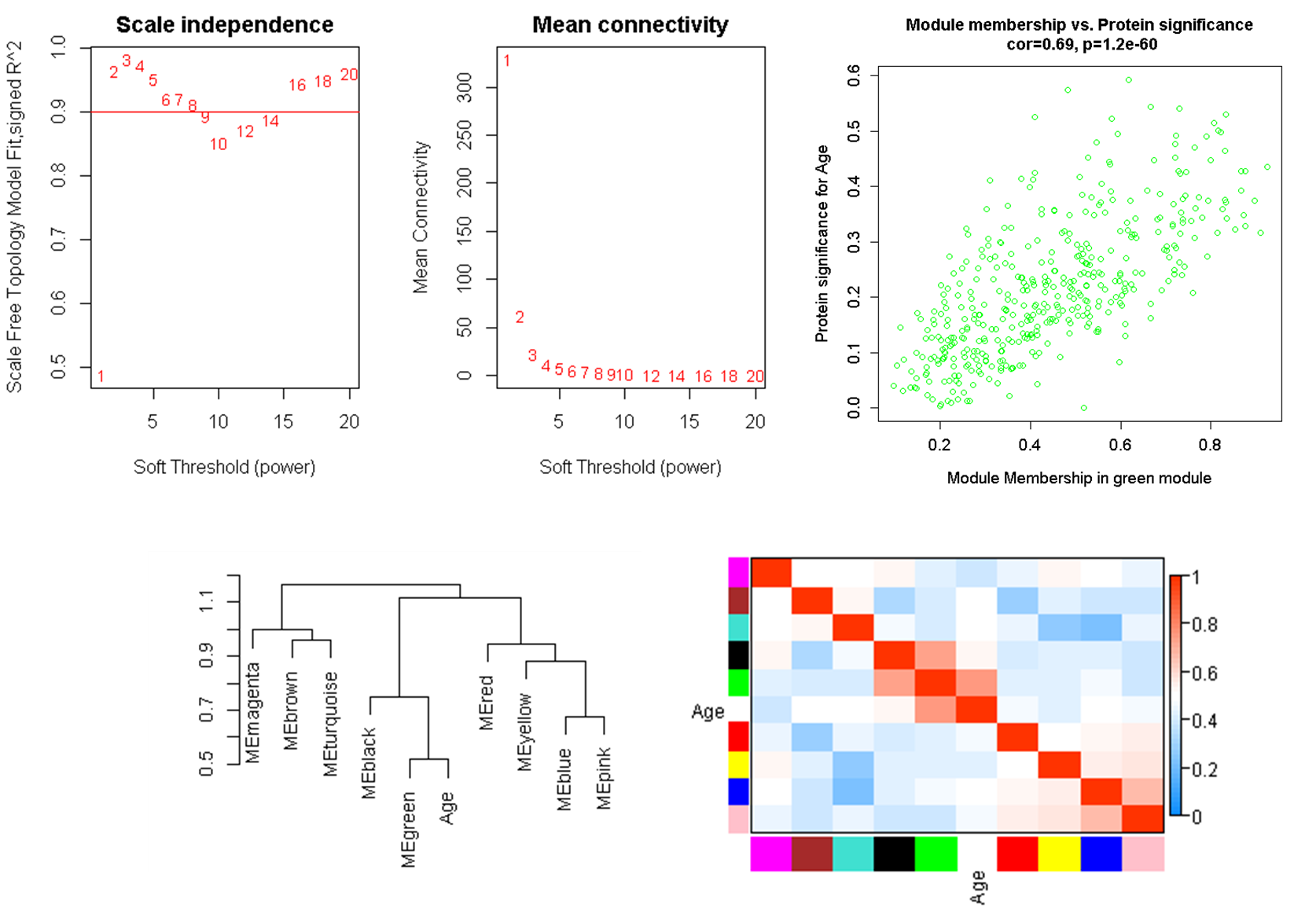


**Supplementary Figure 4: Comparison of Age prediction model derived from 4265 proteins as well as from top 200,100 and 50 proteins associated with Chronological age**


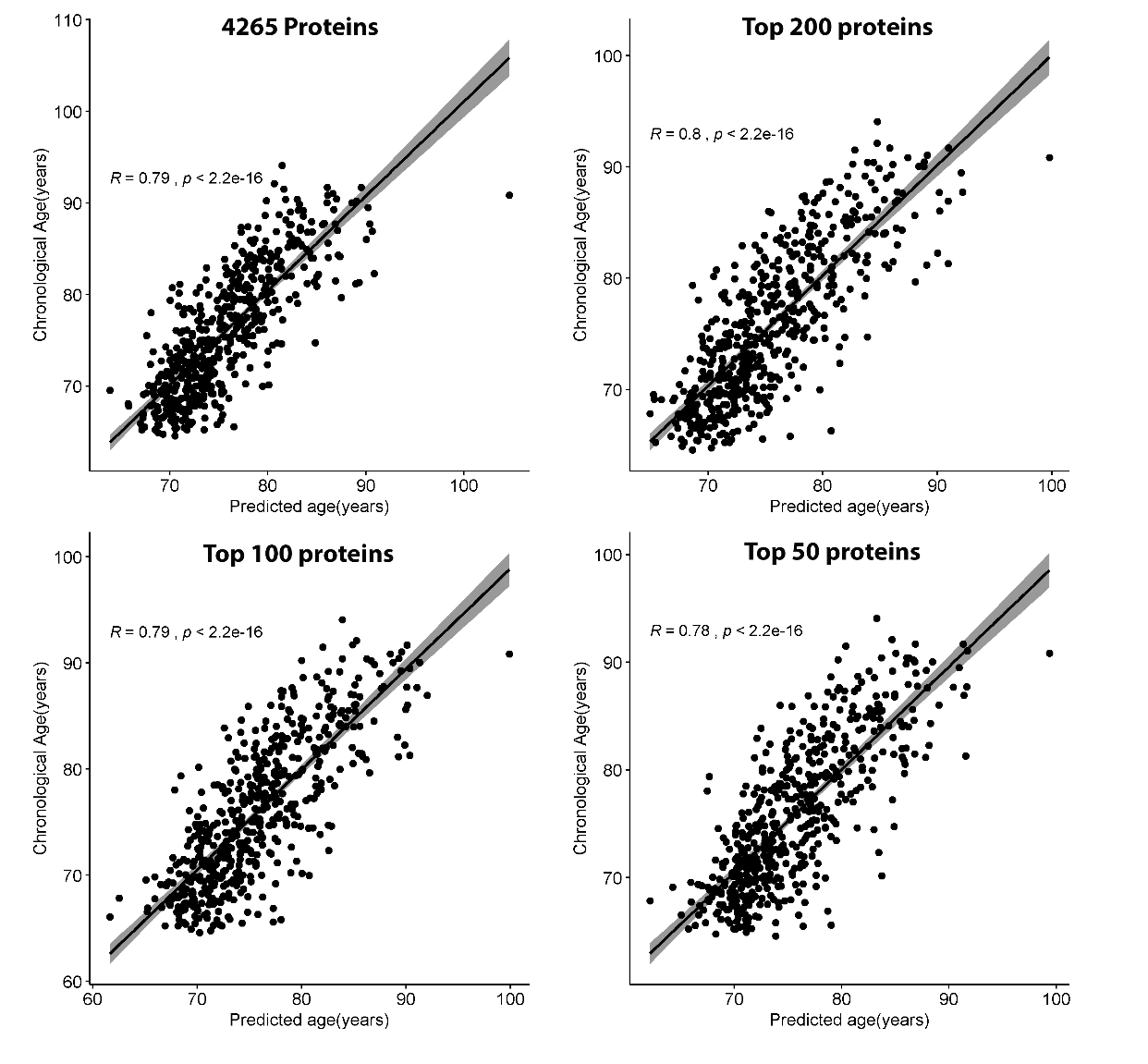


162,74,67 and 35 proteins were selected using elastic net regression for prediction from 4265(total proteins), top 200,100 and 50 age associated proteins respectively

**Supplementary Table 1: Top 30 most significant SOMAmers associated with chronological age in OPEL.**

| Target | Target Full Name | UniProt | Estimate | Std_Error | p value |
| --- | --- | --- | --- | --- | --- |
| PTN | Pleiotrophin | P21246 | 0.0261 | 0.0019 | 1.66E-37 |
| WISP-2 | WNT1-inducible-signaling pathway protein 2 | O76076 | 0.0181 | 0.0014 | 4.31E-35 |
| RSPO1 | R-spondin-1 | Q2MKA7 | 0.0202 | 0.0016 | 6.11E-32 |
| TAGL | Transgelin | Q01995 | 0.0207 | 0.0017 | 5.68E-31 |
| CRDL1 | Chordin-like protein 1 | Q9BU40 | 0.0183 | 0.0015 | 8.86E-31 |
| MIC-1 | Growth/differentiation factor 15 | Q99988 | 0.0269 | 0.0022 | 2.56E-30 |
| ERBB1 | Epidermal growth factor receptor | P00533 | -0.0113 | 0.0010 | 5.67E-28 |
| HE4 | WAP four-disulfide core domain protein 2 | Q14508 | 0.0196 | 0.0017 | 1.04E-27 |
| RNase 1 | Ribonuclease pancreatic | P07998 | 0.0320 | 0.0028 | 3.58E-27 |
| FBLN3 | EGF-containing fibulin-like extracellular matrix protein 1 | Q12805 | 0.0125 | 0.0011 | 1.03E-25 |
| SMOC1 | SPARC-related modular calcium-binding protein 1 | Q9H4F8 | 0.0097 | 0.0009 | 6.20E-25 |
| Cystatin C | Cystatin-C | P01034 | 0.0124 | 0.0012 | 3.60E-23 |
| PGD2 synthase | Prostaglandin-H2 D-isomerase | P41222 | 0.0151 | 0.0015 | 1.91E-22 |
| RSPO3 | R-spondin-3 | Q9BXY4 | 0.0095 | 0.0010 | 7.19E-21 |
| Macrophage scavenger receptor | Macrophage scavenger receptor types I and II | P21757 | 0.0184 | 0.0019 | 1.52E-20 |
| sTREM-1 | Triggering receptor expressed on myeloid cells 1 | Q9NP99 | 0.0170 | 0.0017 | 1.63E-20 |
| a2-Antiplasmin | Alpha-2-antiplasmin | P08697 | -0.0072 | 0.0008 | 3.05E-20 |
| HSPB6 | Heat shock protein beta-6 | O14558 | 0.0182 | 0.0019 | 7.49E-20 |
| SREC-II | Scavenger receptor class F member 2 | Q96GP6 | 0.0086 | 0.0009 | 1.33E-19 |
| URB | Coiled-coil domain-containing protein 80 | Q76M96 | 0.0124 | 0.0013 | 6.28E-19 |
| SAP3 | Ganglioside GM2 activator | P17900 | 0.0114 | 0.0012 | 6.44E-19 |
| ATS13 | A disintegrin and metalloproteinase with thrombospondin motifs 13 | Q76LX8 | -0.0134 | 0.0015 | 1.78E-18 |
| PXDN | Peroxidasin homolog | Q92626 | 0.0208 | 0.0023 | 1.88E-18 |
| CDCP1 | CUB domain-containing protein 1 | Q9H5V8 | 0.0193 | 0.0022 | 5.46E-18 |
| FSTL3 | Follistatin-related protein 3 | O95633 | 0.0118 | 0.0013 | 5.55E-18 |
| Troponin T | Troponin T, cardiac muscle | P45379 | 0.0153 | 0.0017 | 7.66E-18 |
| ASB9 | Ankyrin repeat and SOCS box protein 9 | Q96DX5 | 0.0187 | 0.0021 | 1.15E-17 |
| FABPA | Fatty acid-binding protein, adipocyte | P15090 | 0.0203 | 0.0023 | 2.61E-17 |
| Cathepsin V | Cathepsin L2 | O60911 | -0.0152 | 0.0017 | 4.68E-17 |
| PCDGA | Protocadherin gamma-A10 | Q9Y5H3 | 0.0106 | 0.0012 | 8.28E-17 |

Model: log(SOMAmer)~ age+gender

**Supplementary Table 2: Top 30 most significant SOMAmers associated with chronological age in OPUS.**

| Target | Target Full Name | UniProt | Estimate | Std Error | p value |
| --- | --- | --- | --- | --- | --- |
| PTN | Pleiotrophin | P21246 | 0.0262 | 0.0016 | 6.19E-50 |
| WISP-2 | WNT1-inducible-signaling pathway protein 2 | O76076 | 0.0195 | 0.0012 | 7.68E-48 |
| CRDL1 | Chordin-like protein 1 | Q9BU40 | 0.0218 | 0.0013 | 1.41E-47 |
| FBLN3 | EGF-containing fibulin-like extracellular matrix protein 1 | Q12805 | 0.0150 | 0.0010 | 1.63E-41 |
| TAGL | Transgelin | Q01995 | 0.0219 | 0.0015 | 1.28E-40 |
| RSPO1 | R-spondin-1 | Q2MKA7 | 0.0212 | 0.0015 | 1.09E-39 |
| ERBB1 | Epidermal growth factor receptor | P00533 | -0.0118 | 0.0008 | 3.24E-38 |
| MIC-1 | Growth/differentiation factor 15 | Q99988 | 0.0279 | 0.0021 | 1.56E-35 |
| FSTL3 | Follistatin-related protein 3 | O95633 | 0.0143 | 0.0011 | 3.04E-34 |
| SMOC1 | SPARC-related modular calcium-binding protein 1 | Q9H4F8 | 0.0108 | 0.0008 | 9.56E-34 |
| PGD2 synthase | Prostaglandin-H2 D-isomerase | P41222 | 0.0168 | 0.0014 | 4.27E-31 |
| Cystatin C | Cystatin-C | P01034 | 0.0142 | 0.0012 | 7.64E-30 |
| URB | Coiled-coil domain-containing protein 80 | Q76M96 | 0.0137 | 0.0011 | 1.24E-29 |
| IGFBP-2 | Insulin-like growth factor-binding protein 2 | P18065 | 0.0296 | 0.0025 | 2.23E-28 |
| N-terminal pro-BNP | N-terminal pro-BNP | P16860 | 0.0547 | 0.0047 | 2.29E-28 |
| HE4 | WAP four-disulfide core domain protein 2 | Q14508 | 0.0186 | 0.0016 | 2.89E-28 |
| Macrophage scavenger receptor | Macrophage scavenger receptor types I and II | P21757 | 0.0201 | 0.0017 | 7.02E-28 |
| b2-Microglobulin | Beta-2-microglobulin | P61769 | 0.0132 | 0.0011 | 7.11E-28 |
| TNF sR-II | Tumor necrosis factor receptor superfamily member 1B | P20333 | 0.0148 | 0.0013 | 1.08E-26 |
| a2-Antiplasmin | Alpha-2-antiplasmin | P08697 | -0.0076 | 0.0007 | 1.59E-26 |
| SREC-II | Scavenger receptor class F member 2 | Q96GP6 | 0.0093 | 0.0008 | 1.41E-25 |
| TNF sR-II | Tumor necrosis factor receptor superfamily member 1B | P20333 | 0.0133 | 0.0012 | 2.74E-25 |
| TMEDA | Transmembrane emp24 domain-containing protein 10 | P49755 | 0.0144 | 0.0013 | 4.46E-25 |
| SVEP1 | Sushi, von Willebrand factor type A, EGF and pentraxin domain-containing protein 1 | Q4LDE5 | 0.0173 | 0.0016 | 5.77E-25 |
| Growth hormone receptor | Growth hormone receptor | P10912 | -0.0194 | 0.0018 | 6.45E-25 |
| SET | Protein SET | Q01105 | -0.0086 | 0.0008 | 7.65E-25 |
| SVEP1 | Sushi, von Willebrand factor type A, EGF and pentraxin domain-containing protein 1 | Q4LDE5 | 0.0156 | 0.0014 | 8.39E-25 |
| sTREM-1 | Triggering receptor expressed on myeloid cells 1 | Q9NP99 | 0.0177 | 0.0016 | 9.15E-25 |
| ASB9 | Ankyrin repeat and SOCS box protein 9 | Q96DX5 | 0.0197 | 0.0018 | 1.56E-24 |
| RNase 1 | Ribonuclease pancreatic | P07998 | 0.0275 | 0.0025 | 1.68E-24 |

Model: log(SOMAmer)~ age+gender

**Supplementary Table 3: Top 30 most significant SOMAmers associated with chronological age in males.**

| Target | Target Full Name | UniProt | Estimate | Std. Error | p value |
| --- | --- | --- | --- | --- | --- |
| PTN | Pleiotrophin | P21246 | 0.0293 | 0.0018 | 2.26E-46 |
| CRDL1 | Chordin-like protein 1 | Q9BU40 | 0.0213 | 0.0014 | 1.45E-43 |
| RSPO1 | R-spondin-1 | Q2MKA7 | 0.0234 | 0.0015 | 1.09E-41 |
| FBLN3 | EGF-containing fibulin-like extracellular matrix protein 1 | Q12805 | 0.0157 | 0.0011 | 1.55E-38 |
| WISP-2 | WNT1-inducible-signaling pathway protein 2 | O76076 | 0.0187 | 0.0013 | 3.35E-37 |
| MIC-1 | Growth/differentiation factor 15 | Q99988 | 0.0311 | 0.0022 | 1.27E-36 |
| Macrophage scavenger receptor | Macrophage scavenger receptor types I and II | P21757 | 0.0233 | 0.0018 | 2.08E-31 |
| ERBB1 | Epidermal growth factor receptor | P00533 | -0.0115 | 0.0009 | 5.60E-30 |
| N-terminal pro-BNP | N-terminal pro-BNP | P16860 | 0.0653 | 0.0054 | 9.17E-30 |
| TAGL | Transgelin | Q01995 | 0.0204 | 0.0017 | 1.19E-29 |
| SREC-II | Scavenger receptor class F member 2 | Q96GP6 | 0.0105 | 0.0009 | 3.56E-29 |
| RET | Proto-oncogene tyrosine-protein kinase receptor Ret | P07949 | -0.0213 | 0.0018 | 1.20E-28 |
| PGD2 synthase | Prostaglandin-H2 D-isomerase | P41222 | 0.0183 | 0.0015 | 2.30E-28 |
| OPG | Tumor necrosis factor receptor superfamily member 11B | O00300 | 0.0194 | 0.0016 | 3.61E-28 |
| Cystatin C | Cystatin-C | P01034 | 0.0147 | 0.0012 | 8.67E-28 |
| SMOC1 | SPARC-related modular calcium-binding protein 1 | Q9H4F8 | 0.0107 | 0.0009 | 1.33E-27 |
| TNF sR-II | Tumor necrosis factor receptor superfamily member 1B | P20333 | 0.0173 | 0.0015 | 2.35E-27 |
| HE4 | WAP four-disulfide core domain protein 2 | Q14508 | 0.0204 | 0.0018 | 2.43E-27 |
| FSTL3 | Follistatin-related protein 3 | O95633 | 0.0143 | 0.0012 | 3.62E-27 |
| IGFBP-2 | Insulin-like growth factor-binding protein 2 | P18065 | 0.0317 | 0.0027 | 4.78E-27 |
| TREM2 | Triggering receptor expressed on myeloid cells 2 | Q9NZC2 | 0.0265 | 0.0023 | 1.25E-26 |
| SET | Protein SET | Q01105 | -0.0094 | 0.0008 | 4.06E-26 |
| URB | Coiled-coil domain-containing protein 80 | Q76M96 | 0.0140 | 0.0012 | 4.46E-26 |
| RNase 1 | Ribonuclease pancreatic | P07998 | 0.0314 | 0.0028 | 7.86E-26 |
| SVEP1 | Sushi, von Willebrand factor type A, EGF and pentraxin domain-containing protein 1 | Q4LDE5 | 0.0175 | 0.0016 | 6.61E-25 |
| TNF sR-II | Tumor necrosis factor receptor superfamily member 1B | P20333 | 0.0152 | 0.0014 | 7.98E-25 |
| b2-Microglobulin | Beta-2-microglobulin | P61769 | 0.0139 | 0.0013 | 1.28E-24 |
| NMB | Neuromedin-B | P08949 | 0.0137 | 0.0013 | 1.46E-24 |
| TMEDA | Transmembrane emp24 domain-containing protein 10 | P49755 | 0.0154 | 0.0014 | 1.80E-24 |
| Apo F | Apolipoprotein F | Q13790 | 0.0246 | 0.0023 | 3.15E-24 |

Model: log(SOMAmer)~ age+ cohort

**Supplementary Table 4: Top 30 most significant SOMAmers associated with chronological age phenotype in females.**

| Target | Target Full Name | UniProt | Estimate | Std Error | p_value |
| --- | --- | --- | --- | --- | --- |
| WISP-2 | WNT1-inducible-signaling pathway protein 2 | O76076 | 0.0191 | 0.0012 | 3.04E-46 |
| TAGL | Transgelin | Q01995 | 0.0221 | 0.0015 | 3.91E-42 |
| PTN | Pleiotrophin | P21246 | 0.0236 | 0.0016 | 2.72E-41 |
| CRDL1 | Chordin-like protein 1 | Q9BU40 | 0.0195 | 0.0014 | 4.46E-37 |
| ERBB1 | Epidermal growth factor receptor | P00533 | -0.0116 | 0.0009 | 1.42E-36 |
| RSPO1 | R-spondin-1 | Q2MKA7 | 0.0187 | 0.0015 | 5.05E-32 |
| SMOC1 | SPARC-related modular calcium-binding protein 1 | Q9H4F8 | 0.0100 | 0.0008 | 1.72E-31 |
| FBLN3 | EGF-containing fibulin-like extracellular matrix protein 1 | Q12805 | 0.0125 | 0.0010 | 1.96E-30 |
| MIC-1 | Growth/differentiation factor 15 | Q99988 | 0.0244 | 0.0020 | 3.48E-30 |
| HE4 | WAP four-disulfide core domain protein 2 | Q14508 | 0.0181 | 0.0015 | 8.09E-29 |
| Cystatin C | Cystatin-C | P01034 | 0.0125 | 0.0011 | 2.54E-26 |
| PGD2 synthase | Prostaglandin-H2 D-isomerase | P41222 | 0.0143 | 0.0013 | 8.72E-26 |
| RNase 1 | Ribonuclease pancreatic | P07998 | 0.0280 | 0.0025 | 1.07E-25 |
| FSTL3 | Follistatin-related protein 3 | O95633 | 0.0124 | 0.0012 | 6.97E-25 |
| HSPB6 | Heat shock protein beta-6 | O14558 | 0.0183 | 0.0017 | 3.65E-24 |
| sTREM-1 | Triggering receptor expressed on myeloid cells 1 | Q9NP99 | 0.0169 | 0.0016 | 9.98E-24 |
| a2-Antiplasmin | Alpha-2-antiplasmin | P08697 | -0.0069 | 0.0007 | 2.45E-23 |
| URB | Coiled-coil domain-containing protein 80 | Q76M96 | 0.0125 | 0.0012 | 6.01E-23 |
| ATS13 | A disintegrin and metalloproteinase with thrombospondin motifs 13 | Q76LX8 | -0.0131 | 0.0013 | 5.98E-22 |
| ASB9 | Ankyrin repeat and SOCS box protein 9 | Q96DX5 | 0.0182 | 0.0019 | 7.33E-21 |
| Troponin T | Troponin T, cardiac muscle | P45379 | 0.0145 | 0.0015 | 2.69E-20 |
| aldolase C | Fructose-bisphosphate aldolase C | P09972 | -0.0127 | 0.0013 | 4.11E-20 |
| SAP3 | Ganglioside GM2 activator | P17900 | 0.0109 | 0.0011 | 5.85E-20 |
| b2-Microglobulin | Beta-2-microglobulin | P61769 | 0.0109 | 0.0012 | 1.65E-19 |
| SLPI | Antileukoproteinase | P03973 | 0.0102 | 0.0011 | 4.48E-19 |
| PXDN | Peroxidasin homolog | Q92626 | 0.0189 | 0.0020 | 4.92E-19 |
| CDON | Cell adhesion molecule-related/down-regulated by oncogenes | Q4KMG0 | -0.0098 | 0.0011 | 5.05E-19 |
| Macrophage scavenger receptor | Macrophage scavenger receptor types I and II | P21757 | 0.0161 | 0.0017 | 6.47E-19 |
| CDCP1 | CUB domain-containing protein 1 | Q9H5V8 | 0.0171 | 0.0019 | 1.09E-18 |
| SREC-II | Scavenger receptor class F member 2 | Q96GP6 | 0.0078 | 0.0009 | 2.18E-18 |

Model: log(SOMAmer)~ age+ cohort

**Supplementary Table 5: Top pathways associated with aging using Reactome database.**

| Pathway name | Entities | | | | Reactions | |
| --- | --- | --- | --- | --- | --- | --- |
|  | found | ratio | p-value | FDR* | found | ratio |
| Regulation of Insulin-like Growth Factor (IGF) transport and uptake by Insulin-like Growth Factor Binding Proteins (IGFBPs) | 38 / 127 | 0.009 | 2.75E-14 | 4.57E-11 | 12 / 14 | 1.00E-03 |
| Extracellular matrix organization | 59 / 329 | 0.023 | 8.15E-12 | 6.76E-09 | 171/318 | 2.60E-02 |
| Post-translational protein phosphorylation | 30 / 109 | 0.008 | 1.02E-10 | 5.65E-08 | 1/1 | 8.19E-05 |
| Degradation of the extracellular matrix | 33 / 148 | 0.01 | 2.26E-09 | 9.34E-07 | 70/105 | 9.00E-03 |
| Response to elevated platelet cytosolic Ca2+ | 29 / 144 | 0.01 | 1.71E-07 | 5.67E-05 | 9/14 | 1.00E-03 |
| Platelet degranulation | 27 / 137 | 0.01 | 6.77E-07 | 1.87E-04 | 6 / 11 | 9.01E-04 |
| Collagen degradation | 17 / 69 | 0.005 | 4.71E-06 | 1.00E-03 | 23 / 34 | 3.00E-03 |
| Formation of Fibrin Clot (Clotting Cascade) | 14 / 55 | 0.004 | 2.23E-05 | 5.00E-03 | 47 / 57 | 5.00E-03 |
| Collagen formation | 20 / 104 | 0.007 | 2.56E-05 | 5.00E-03 | 42 / 77 | 6.00E-03 |
| Neutrophil degranulation | 55 / 480 | 0.034 | 5.48E-05 | 8.00E-03 | 10 / 10 | 8.19E-04 |
| Collagen biosynthesis and modifying enzymes | 16 / 76 | 0.005 | 5.71E-05 | 8.00E-03 | 33 / 51 | 4.00E-03 |
| Hemostasis | 83 / 820 | 0.058 | 6.10E-05 | 8.00E-03 | 139 /327 | 2.70E-02 |
| Signaling by FGFR1 | 14 / 61 | 0.004 | 6.69E-05 | 8.00E-03 | 45 / 48 | 4.00E-03 |
| Negative regulation of FGFR1 signaling | 11 / 39 | 0.003 | 6.77e-05 | 8.00E-03 | 14 / 15 | 0.001 |

**Reactome**defines a 'reaction' as any event in biology that changes the state of a biological molecule. Binding, activation, translocation, degradation and classical biochemical events involving a catalyst are all reactions.

**Supplementary Table 6:** Top IPA Bio functions and disease pathway associated with aging. IPA generated associated networks which are ordered by a score denoting significance.

| **Top Diseases and Bio Functions** |  |  |
| --- | --- | --- |
| **Diseases and Disorders** |  |  |
| **Name** | **p-value range** | **#Molecules** |
| Cancer | 4.05E-09 - 5.83E-36 | 702 |
| Organismal Injury and Abnormalities | 4.05E-09 - 5.83E-36 | 712 |
| Inflammatory Response | 3.54E-09 - 9.48E-34 | 322 |
| Reproductive System Disease | 2.31E-09 - 4.94E-24 | 455 |
| Connective Tissue Disorders | 3.54E-09 - 4.66E-23 | 220 |
|  |  |  |
| **Molecular and Cellular Functions** |  |  |
| **Name** | **p-value range** | **#Molecules** |
| Cellular Movement | 3.51E-09 - 1.04E-43 | 296 |
| Cell Death and Survival | 3.97E-09 - 8.33E-33 | 324 |
| Cell-To-Cell Signaling and Interaction | 2.86E-09 - 1.75E-32 | 280 |
| Cellular Compromise | 2.53E-10 - 1.05E-22 | 102 |
| Cellular Growth and Proliferation | 2.92E-09 - 6.81E-18 | 332 |
|  |  |  |
| **Physiological System Development and Function** |  |  |
| **Name** | **p-value range** | **#Molecules** |
| Immune Cell Trafficking | 3.51E-09 - 1.98E-39 | 196 |
| Hematological System Development and Function | 3.51E-09 - 2.24E-39 | 276 |
| Tissue Morphology | 3.13E-09 - 3.12E-30 | 269 |
| Cardiovascular System Development and Function | 2.72E-09 - 4.44E-30 | 211 |
| Organismal Survival | 1.36E-20 - 3.03E-29 | 265 |
|  |  |  |
| **Associated Network Functions** | **Score** |  |
| Cellular Movement, Cellular Development, Cellular Growth and Proliferation | 41 |  |
| Cellular Movement, Nervous System Development and Function, Cell-To-Cell Signaling and Interaction | 37 |  |
| Cancer, Cellular Development, Cellular Growth and Proliferation | 35 |  |
| Hematological System Development and Function, Organismal Functions, Cardiovascular Disease | 33 |  |
| Connective Tissue Development and Function, Connective Tissue Disorders, Developmental Disorder | 33 |  |

**Supplementary Table 7:** Top IPA Bio functions and disease pathway associated with green module in WGCNA. IPA generated associated networks which are ordered by a score denoting significance.

| **Top Diseases and Bio Functions** | | |
| --- | --- | --- |
| **Diseases and Disorders** | | |
| Name | p-value | #Molecules |
| Inflammatory Response | 1.01E-06 - 2.61E-28 | 197 |
| Cancer | 1.00E-06 - 2.39E-22 | 389 |
| Organismal Injury and Abnormalities | 1.00E-06 - 2.39E-22 | 396 |
| Connective Tissue Disorders | 5.41E-07 - 9.07E-18 | 109 |
| Inflammatory Disease | 9.18E-07 - 9.07E-18 | 134 |
|  |  |  |
| Molecular and Cellular Functions |  |  |
| Cellular Movement | 9.38E-07 - 3.67E-34 | 185 |
| Cell-To-Cell Signaling and Interaction | 1.01E-06 - 6.80E-30 | 170 |
| Cell Death and Survival | 9.74E-07 - 7.60E-25 | 194 |
| Cellular Development | 9.72E-07 - 3.68E-21 | 190 |
| Cellular Growth and Proliferation | 9.72E-07 - 3.68E-21 | 198 |
|  |  |  |
| Physiological System Development and Function |  |  |
| Hematological System Development and Function | 1.01E-06 - 3.67E-34 | 178 |
| Immune Cell Trafficking | 1.01E-06 - 3.67E-34 | 131 |
| Tissue Morphology | 8.03E-07 - 5.66E-24 | 163 |
| Lymphoid Tissue Structure and Development | 8.03E-07 - 1.20E-19 | 128 |
| Cardiovascular System Development and Function | 9.50E-07 - 1.12E-15 | 114 |
|  |  |  |
| Top Networks |  |  |
| Associated Network Functions | Score |  |
| Cellular Movement, Immune Cell Trafficking, Cell-To- Cell Signaling and Interaction | 44 |  |
| Cell-To-Cell Signaling and Interaction, Cell Death and Survival, Cell Cycle | 39 |  |
| Hematological System Development and Function, Lymphoid Tissue Structure and Development, Tissue Morphology | 37 |  |
| Cancer, Endocrine System Disorders, Gastrointestinal Disease | 35 |  |
| Tissue Development, Connective Tissue Disorders, Organismal Injury and Abnormalities | 33 |  |

**Supplementary Table 8:** Top IPA Bio functions and disease pathway associated with magenta module in WGCNA. IPA generated associated networks which are ordered by a score denoting significance.

| **Top Diseases and Bio Functions** | | |
| --- | --- | --- |
| **Diseases and Disorders** | | |
| **Name** | **p-value** | **#Molecules** |
| Cancer | 2.56E-02 - 5.81E-07 | 31 |
| Gastrointestinal Disease | 2.56E-02 - 5.81E-07 | 29 |
| Hepatic System Disease | 2.56E-02 - 5.81E-07 | 21 |
| Organismal Injury and Abnormalities | 2.56E-02 - 5.81E-07 | 31 |
| Hereditary Disorder | 2.43E-02 - 7.45E-05 | 18 |
|  |  |  |
| **Molecular and Cellular Functions** |  |  |
| **Name** | **p-value** | **#Molecules** |
| Energy Production | 2.29E-02 - 2.00E-09 | 8 |
| Small Molecule Biochemistry | 2.29E-02 - 2.00E-09 | 23 |
| Drug Metabolism | 1.76E-02 - 3.89E-07 | 7 |
| Lipid Metabolism | 2.29E-02 - 1.78E-06 | 13 |
| Vitamin and Mineral Metabolism | 1.89E-02 - 1.78E-06 | 8 |
|  |  |  |
| **Physiological System Development and Function** |  |  |
| **Name** | **p-value** | **#Molecules** |
| Endocrine System Development and Function | 1.36E-02 - 3.72E-04 | 3 |
| Digestive System Development and Function | 2.56E-02 - 1.36E-03 | 7 |
| Organ Morphology | 2.43E-02 - 1.36E-03 | 4 |
| Organismal Development | 2.43E-02 - 1.36E-03 | 6 |
| Respiratory System Development and Function | 1.36E-03 - 1.36E-03 | 1 |
|  |  |  |
| **Top Networks** |  |  |
| **Associated Network Functions** | **Score** |  |
| Energy Production, Small Molecule Biochemistry, Organismal Injury and Abnormalities | 47 |  |
| Lipid Metabolism, Small Molecule Biochemistry, Cellular Compromise | 31 |  |

**Supplementary Methods**

Frailty: The two common approaches adopted to define frailty clinically are as a cumulative deficit index ^1,2^ or as a clinical syndrome^3^. In the present study we have used the cumulative deficit index proposed by Rockwood *et al.* ^4^. The variables selected for the frailty index (FI) construction were based on standardized criteria that includes: association with health status, biologically relevant, accumulates with age, and must represent multiple organ systems^4^. Further variables should not saturate early with age like presbyopia, which are quite common by age 55 and are excluded. A minimum of 30 variables is recommended for developing the FI ^2^, and has been shown to predict deteriorating health status, institutionalization, and death ^2^. Based on the recommended approach 41 variables were included in the present study ^2^. In case of binary variables, 0 represent no deficit and 1 represents a deficit. Continuous or rank variables were graded from 0 (no deficit) to 1 (maximum deficits). The variables and cut-off used for construction of frailty index are shown in Table 1. The FI was calculated by adding the number of deficits (value=1) and dividing the total by the total number of variables per participant; resulting in a range of scores from 0 to 1 for each individual ^2^.

**Table. Health variables used for construction of cumulative frailty index**

| Sl. No. | Variables | Coding |
| --- | --- | --- |
| 1 | Help bathing | Yes = 1, No = 0 |
| 2 | Help dressing | Yes = 1, No = 0 |
| 3 | Help getting in/out of chair | Yes = 1, No = 0 |
| 4 | Help walking around house | Yes = 1, No = 0 |
| 5 | Help eating | Yes = 1, No = 0 |
| 6 | Help grooming | Yes = 1, No = 0 |
| 7 | Help using toilet | Yes = 1, No = 0 |
| 8 | Help up/down stairs | Yes = 1, No = 0 |
| 9 | Help lifting 10 lb | Yes = 1, No = 0 |
| 10 | Help shopping | Yes = 1, No = 0 |
| 11 | Help with housework | Yes = 1, No = 0 |
| 12 | Help with meal preparations | Yes = 1, No = 0 |
| 13 | Help taking medication | Yes = 1, No = 0 |
| 14 | Help with finances | Yes = 1, No = 0 |
| 15 | Lost more than 10 lb in last year | Yes = 1, No = 0 |
| 16 | Self rating of health | Poor = 1, Fair = 0.75, Good = 0.5, Very Good = 0.25, Excellent = 0 |
| 17 | How health has changed in last year | Worse = 1, Better/Same = 0 |
| 18 | Hospitalized/ER visits | Yes = 1, No = 0 |
| 19 | Cut down on usual activity (in last month) | Yes = 1, No = 0 |
| 20 | Walk outside | <3 days = 1, ≥3 days = 0 |
| 21 | Feel everything is an effort | Most of time = 1, Sometimes = 0.5, Rarely = 0 |
| 22 | Feel depressed | Most of time = 1, Sometimes = 0.5, Rarely = 0 |
| 23 | Feel happy | Most of time = 0, Sometimes = 0.5, Rarely = 1 |
| 24 | Health interfered with social activities | Not at all - Slightly = 0, Moderately - Extremely = 1 |
| 25 | Have trouble getting going | Most of time = 1, Sometimes = 0.5, Rarely = 0 |
| 26 | Moderate activity affected | Yes = 1, No = 0 |
| 27 | High blood pressure | Yes = 1, No = 0 |
| 28 | Heart attack | Yes = 1, No = 0 |
| 29 | CHF | Yes = 1, No = 0 |
| 30 | Stroke | Yes = 1, No = 0 |
| 31 | Cancer | Yes = 1, No = 0 |
| 32 | Diabetes | Yes = 1, No = 0 |
| 33 | Arthritis | Yes = 1, No = 0 |
| 34 | Chronic Lung Disease | Yes = 1, No = 0 |
| 35 | Cognitive test: Blessed | <2=0: 2-3=0.25: 4-7=0.50: >7 =1 |
| 36 | Peak flow | 1 if Men=<340 liters/min; Women=<310 liters/min |
| 37 | BMI | 1 if <18.5 or >=30 |
| 38 | Grip strength | 1 if Men BMI=<24, GS=<29 :Men BMI 24.1-28, GS=<30:Men BMI>28, GS=<32:  Women BMI=<23, GS=<17:Women BMI 23.1-26, GS=<17.3:Women BMI 26.1-29,GS=<18: Women BMI >29, GS=<21 |
| 39 | Falls | Yes = 1, No = 0 |
| 40 | Memory changes | Yes = 1, No = 0 |
| 41 | History of Parkinson's disease | Yes = 1, No = 0 |

1. Rockwood, K., Mogilner, A. & Mitnitski, A. Changes with age in the distribution of a frailty index. *Mechanisms of ageing and development* **125**, 517-519 (2004).

2. Rockwood, K. & Mitnitski, A. Frailty in relation to the accumulation of deficits. *The Journals of Gerontology Series A: Biological Sciences and Medical Sciences* **62**, 722-727 (2007).

3. Fried, L.P. *et al.* Frailty in older adults evidence for a phenotype. *The Journals of Gerontology Series A: Biological Sciences and Medical Sciences* **56**, M146-M157 (2001).

4. Searle, S.D., Mitnitski, A., Gahbauer, E.A., Gill, T.M. & Rockwood, K. A standard procedure for creating a frailty index. *BMC geriatrics* **8**, 24 (2008).
